# Multiple Sclerosis in the Emirati Population: Onset Disease Characterization by MR Imaging

**DOI:** 10.1155/2019/7460213

**Published:** 2019-11-25

**Authors:** Manzoor Ahmed, Ruqqiya Mir, Mustafa Shakra, Safana Al Fardan

**Affiliations:** ^1^Department of Radiology, Sheikh Khalifa Medical City, Abu Dhabi, UAE; ^2^Department of Neurology, Sheikh Khalifa Medical City, Abu Dhabi, UAE; ^3^Department of Psychiatry, Sheikh Khalifa Medical City, Abu Dhabi, UAE

## Abstract

**Background and Objectives:**

Multiple Sclerosis (MS) epidemiology is on the path of globalization mainly due to changing environmental factors. The prevalence of MS is on the rise in the Middle East and Persian Gulf region. Our observations has led us to hypothesize a heavy MRI lesion load at the onset of disease in a relatively younger native population. We aimed to estimate and characterize the onset disease on MRI using McDonald's criteria while applying its terms of “Dissemination in Space (DIS) and Dissemination in Time (DIT)”.

**Materials and Methods:**

Retrospective review of onset MRI studies of 181 Emirati (native) individuals. Basic demographics were captured. Only 47 patients with Clinically Definite MS (CDMS) were included who had onset diagnostic MRI available. Lesion load was quantified using the specific zones of involvement designated for DIS: (1) Periventricular (PVZ) (I), (2) Juxta-cortical (II) (3) Infra-tentorial (III) and, (4) Spinal cord (IV). PVZ was sub-classified and lesions were quantified. A single enhancing lesion was required for DIT.

**Results:**

Average age of onset was about 26 years with female dominance of about 2 : 1. About 50% had all 4 zones and about 85% had at least 3 zones involved at the onset. Involvement of only 1 zone was rare. Dissemination in time (DIT) in brain and/or cord was present in approximately 50%. Each of the 4 zones were involved in at least 70% of cases. PVZ was not spared in any case with at least 3 lesions present in approx. 95% and ≥12 lesions in approx. half of the patients. Spinal cord specifically cervical cord was involved in up to 80% with typical patchy lesions.

**Conclusion:**

Onset disease characterization using MRI in a young Emirati cohort showed a heavy lesion load in the brain and spinal cord at the onset, signifying cumulative disease before presentation. Disseminated disease also facilitated early diagnosis of MS. The findings have significant potential ramifications for local environmental and cultural factors, as well as disease course and disability progression.

## 1. Introduction

The incidence and prevalence of Multiple Sclerosis (MS) is on the rise globally in nontemperate regions [1–3]. This trend is more notable in the Gulf Region of the Middle East, paradoxical to its hot and dry climate. It is primarily blamed on the limited exposure to sunlight, with the average individual having an increased exposure to cooler, air-conditioned, indoor atmospheres. Research has shown that Vitamin D deficiency is a risk factor for MS [4]. There is clear evidence in recent literature regarding the rising trend of MS epidemiology in the region [5].

A study conducted by one of the major health systems within the UAE (in the Emirate of Abu Dhabi) by Scheiss et al. [6] showed a moderate prevalence of approx. 64/100,000 (age standardized), which is almost double the global average. More than 60% of the patients in the study represented the native (Emirati) population. There are other studies highlighting the increasing prevalence of MS in this region [7, 8]. There are also higher incidences reported in pediatric and adolescent age groups in the UAE [9].

We hereby focus on the qualification and quantification of the disease in the local population at the onset, as manifested on conventional MR imaging. Given the inception of MS during early adulthood (usually when patients are in their 20 s and 30 s), or in some cases during adolescence followed by a long-standing albeit disabling clinical course, the initial imaging is expected to show low burdens of disease with fewer plaques. We have observed in this region that most of the young patients harbor a much higher load of disease, at the onset. Therefore, we hypothesized that MS in the local Emirati population tends to present clinically after cumulation of considerable load of disease. The terminology and criteria used in 2010 McDonald's criteria was used i.e Dissemination in Space (DIS) and Dissemination in Time (DIT) [10–12].

## 2. Material & Methods

This retrospective chart review study was approved by the IRB. The local database of MS patients was utilized. The following inclusion criteria for MS patients and onset MRI examinations were used:

- Local (Emirati) patients only.
- Patients with eventual clinically definite MS (CDMS).
- MS cases with available first diagnostic/onset MRI brain with or without Gadolinium injection.
- Onset MRI Cervical and/or thoracic spine (if available).

The exclusion criteria included:

- Non-Emiratis (mostly expatriates).
- Patients with ambiguous date of onset or CDMS diagnosis.
- Patients with Clinical Isolated Syndrome (CIS) or Radiologic Isolated Syndrome (RIS) without later conversion, and Neuromyelitis Optica Syndrome.
- Technically limited MRI examinations (especially outside hospital limited or incomplete scans).

The chart review in the electronic medical records (EMR) was used to capture basic demographics of the patients. The onset presentation was confirmed in addition to the corresponding onset MRI, as these were the two crucial components of this study.

As the MRI examinations included both inside and outside studies, protocol control was not possible. Axial 5 mm T2 FLAIR, FSE T2, Sagittal FLAIR or T2 were mandatory sequences which constituted conventional MRI MS protocol examinations. Some patients had volume acquisition, modern protocols with axial reconstructions. More than 5 minutes delay was required for post-contrast axial T1 imaging.

MRI studies were reviewed by a board-certified neuro-radiologist (MA) for onset disease characterization (ODC). The ODC had two components:

*(i) Lesion Load Dissemination (Qualification)*. The approach followed the McDonald Diagnostic criteria. The criteria have recently been modified (in 2017) but in this report the authors used the 2010 criteria. Dissemination in Space (DIS), or accumulation of lesions in space, means there is at least 1 MS type T2 lesion in at least two of the following four CNS locations or zones: (1) Periventricular zone (PVZ), (2) Juxta-cortical, (3) Infra-tentorial fossa, (4) Spinal cord. These zones were then used to measure the disease load.

Dissemination in time (DIT) or accumulation of lesions over time is defined as an asymptomatic, enhancing lesion plus one T2 lesion or a new lesion, on follow up exam. We used at least 1 enhancing brain or cord lesion to qualify for DIT at the onset.

*(ii) Lesions Load Estimation (Quantification)*. The 4 zones were used to quantify DIS at the onset based on how many zones were involved. PVZ was further classified into calloso-septal interface and anterior temporal periventricular lesions, given their relative specificity for MS. As lesions have a high preponderance in PVZ and because an increase in number increases specificity, the lesions in PVZ were counted and sub-classified. Quantification of DIT was not needed as only a single enhancing lesion is required for diagnosis.

## 3. Results

A total of 181 Emirati MS patients were identified. However, only 47 of them met the inclusion criteria for the first diagnostic MRI Brain with or without MRI spine available for review. There was female dominance over males with a ratio of about 1.9 in the total pool of 181 patients.

The average age of onset was about 26 years. Age range-based distribution was 15–20 years (32%), 21–30 years (43%) and 31–40 years (23%). About 75% were less than 30 years of age.

Zonal-based dissemination in space (DIT) is shown in Table 1. About 50% had all 4 zones and about 85% had at least 3 zones involved at the onset. Almost all of them met the McDonald criteria of DIS (needing at least 1 lesion in 2 zones). Involvement of only 1 zone was rare. Dissemination in time (DIT) in brain and/or cord was present in about 50% of patients on the first MRI examination.

Elaborated onset DIS is shown in Table 2. The average largest lesion was approx. 11 mm.

Each zone was involved in more than 70% of patients. As expected, PVZ was involved in all cases. Few cortical lesions were detected in addition to the juxta-cortical lesions seen in most patients. Regarding spinal cord lesions, cervical cord lesions were present in approx. 80% of patients and the lesions had typical, peripheral, small patchy pattern. However, about 30% of the lesions were short and multi-segmental (up to 2 segments in the cranio-caudal dimension). None of the cases had longitudinally extensive transverse myelitis (LETM) type of lesions (≥3 segments long). About 40% had at least 3 or more lesions. Optic nerves were not specifically imaged in any of the cases.

The lesion load, as expected, was highest in the PVZ (I) with involvement in 100% of patients at the onset. About half of them also had a very high load of 12 or more periventricular lesions. About 75% had at least 6 lesions, and up to 95% had at least 3 lesions in the PVZ (Table 3).

## 4. Discussion

This study of Onset Disease Characterization (ODC) of MS on conventional MRI, targeting only the local Emirati population, yielded results comparable to the pre-test observations. The Emirati MS cohort presented at a younger age with a much higher lesion load. The lesion load was characterized (both quantification and qualification) using the McDonald criteria terms of Dissemination in Space and Time (DIS and DIT). This is a convenient, reproducible, and measurable approach based on well-established concepts in the MR imaging of MS.

The basic demographics of the patients, in terms of younger age of onset (average about 26 years) and a higher female ratio (F : M-2 : 1), was comparable to the global trend across the literature [2]. About 90% of the patients were in the age range of 20–50 years old. Up to 80% of the patients had relapsing remitting MS (RRMS). Oral disease modifying therapies (DMT) were most commonly used, followed by injectables and infusions. Thyroid disorders and depression were the most common co-morbidities. As environmental factors have been incriminated in the pathogenesis of the disease [4, 13, 14] (although there are some reservations regarding this [15]), exposure to these extrinsic factors at a very young age must play a role in this region. There have been shifting trends in lifestyles of the local population: decreased exposure to sun-light (and therefore Vitamin D deficiency), cumulative increase in exposure to air-conditioned cooler temperatures throughout the year, and the adoption of lifestyles similar to those found in the Western World where MS has typically been more prevalent. Vitamin deficiency (which has been regarded by some to be a global epidemic) is quite prevalent in the UAE primarily due to the hot climate (resulting in indoor life activities). It is further exacerbated due to the cultural and religious customs of full body covered clothing, especially in females [16, 17].

**Figure 1:**
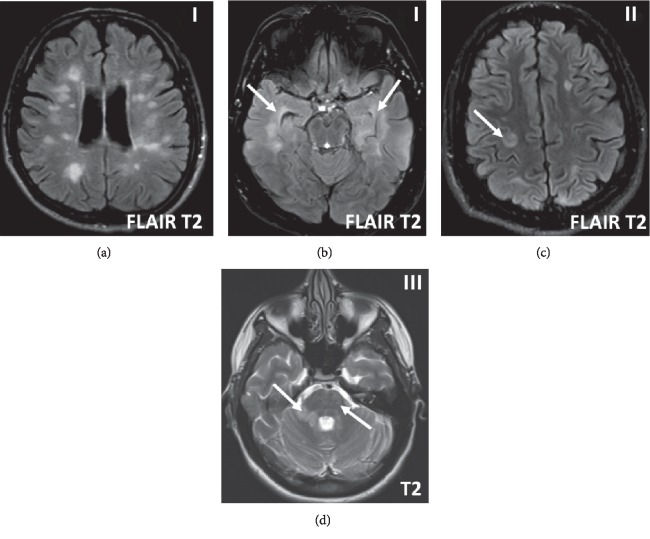
Onset disease axial FLAIR & T2 MR brain imaging. Heavy Periventricular zone (PVZ-I) lesion load (a & b). Note specific anterior temporal PVZ lesions (b, arrows). Cortical-juxta-cortical (zone II) lesion (c, arrow), other lesions are sub-cortical. Infratentorial (zone III) lesions in pons (d, subtle) and brachium pontis (large lesion right side (d, arrow).

**Figure 2:**
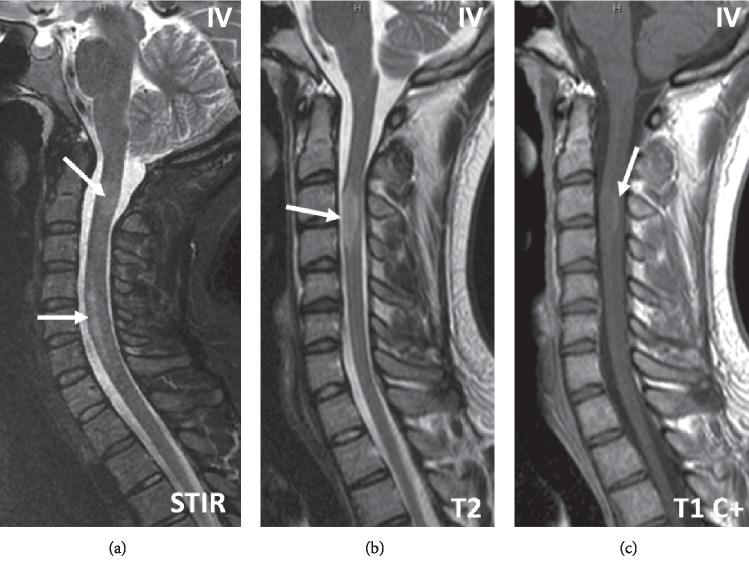
Onset disease Sagittal STIR, T2 & enhanced T1 MR spinal cord imaging. Multiple patchy cervical cord lesions (a, arrows). Note confluent lesions lower cervical cord. Single multi-segmental elongated lesion (b, arrow) with rim enhancement (c, arrow).

There was widespread T2 lesion load at the onset in most patients. Almost all the patients had at least 1 lesion in at least 2 of the zones thus meeting the McDonald criteria of DIS (Figures 1 and 2). As these patients present with a first attack in the form of Clinical Isolated Syndrome (CIS), most of them will only need oligoclonal bands in the CSF (as a marker of relapsing disease and alternative to DIT) to be diagnosed with clinically definite MS (CDMS) according to the 2017 revised McDonald criteria [12]. In fact, about 85% had 3 and 50% had at least 4 zone involvement at the onset. There is retrospective evidence since the 2017 revision of the McDonald criteria, that has shown a higher diagnostic conversion of the patients to definite MS (ranging from 68 to 100%), when compared with the 2005 and 2010 criteria [18]. Pooled analysis from 5 studies showed diagnosis of MS in about 68% using the 2017 criteria compared to about 37% using 2010 criteria [18]. Oligoclonal bands play a major role in this conversion, due to the change in the diagnostic criteria [18]. There is some compromise of the specificity using the 2017 criteria. However, specific distribution and morphology of the lesions can add specificity to the lesions e.g. callosal peri-callosal, brachium pontis or anterior temporal locations as well as features on higher field MRI like central vein sign, rim sign and leptomeningeal enhancement [19–21]. As we used conventional MR Imaging of MS, we only addressed the lesions based on their specific locations.

The two major findings of our study comprised of younger onset age and heavy onset MRI dissemination. The younger age reflects the predominant younger composition of the population like other developing countries. There is a pathophysiological linkage with environmental factors, as alluded to above, with a noteworthy observation of preferential adoption of modern western-style lifestyles by the younger population.

Each zone of DIS was involved at the onset in at least 70% of the patients. The PVZ was not spared in any of the patients. More specifically, the callosal/calloso-septal interface and anterior temporal periventricular zones were involved in more than 75% of the cases. These are well known and hallmark specific regions of involvement, by MS in addition to “Dawson fingers” and brachium pontis lesions, which differentiate them from vascular lesions. They lend specificity and confidence to the true diagnosis of MS patients, at the onset [22]. About 95% of them had at least 3 or more lesions in the PVZ. This is significant, as the MAGNIMS (Magnetic Resonance imaging in MS-European MS Network) recommended in 2016 that at least 3 lesions in PVZ are required instead of 1 lesion for DIS, given the non-specificity and common occurrence of periventricular lesions in non-MS patients [23]. However, this was not adopted in the 2017 McDonald criteria.

DIS in brainstem or cerebellum (Zone III) and cervical cord (Zone IV) ranged from 70% to 80%, implying the true diagnosis of the disease given its specificity in these zones. The morphology of the lesions was a typical patchy distribution (Figure 2(a)). About 2/5^th^ of the patients had at least 3 cervical cord lesions identified, and in some cases, there was diffuse extensive disease. No unusual elongated lesions were identified, specifically longitudinal extensive transverse myelitis (LETM) type lesions as seen in neuromyelitis optica spectrum disorders (NMOSD). There were two patients that had 2 segments long lesions (Figures 2(b) and 2(c)). Interestingly, about 2/3^rd^ of the cases had at least a single discernible lesion in the thoracic cord (in patients who had an initial MRI thoracic spine).

DIS in the juxta-cortical zone was high as well. This zone is gaining more significance as higher field MRI is revealing an increased disease burden with incorporation of cortical lesions in this zone, in the 2017 criteria (Figure 1(d)). Furthermore, there has been more detailed topography of these lesions as manifested on high field MRI namely cortical, leuko-cortical and juxta-cortical. Cortical lesions are further sub-classified into sub-pial, intra-cortical and trans-cortical [24].

DIT at the onset in brain and/or cord was somewhat high and presented in about half of the patients. This can help in the initial diagnosis of MS, as even symptomatic lesions are now included in the count. However, given such a heavy DIS at the onset in patients in this study, MS diagnosis can even be made without the injection of Gadolinium as the 2017 criteria is now more flexible by using oligoclonal bands as a marker of DIT.

The predictive role of MRI in MS is generally twofold: (a) predicting conversion of CIS or RIS to CDMS (the high burden of T2 lesions in this report will have a high predictive value), (b) predicting disability is multifactorial including lesion load at the onset which can be used in the longitudinal study in Emirati MS patients. Besides MR predictive value in conversion and disability, it is important to address the role of treatment in such a cohort with an advanced burden of disease. The time-tested approach of “treat early” until a certain point of disability is being challenged [25]. The modifying therapy may still modify the disease despite advanced disability. Therefore, cohorts of patients like our study can benefit from early treatment. Its important to note that heavy burden of lesions on imaging at the onset is not the only factor of disability to preclude early treatment with DMT.

There were 4 limitations the authors identified in this study. First, it was a retrospective study. Second, there was a relatively small number of eligible patients, as it was subjected to availability of diagnostic MRI with strict inclusion criteria of true onset MRI exams (subjected to delayed referral and archival loss of exams). Thirdly, the MRI exams included in the study mostly used conventional protocols without thin slice volume acquisitions in all cases and 1.5 T MRI was used for many exams. This limited optimal delineation of the juxta-cortical zone from sub-cortical lesions in some cases. Lastly, there is a possibility of selection bias as the authors selected cases of CDMS and excluded cases with CIS where the patient did not convert. However, one can present a counter argument, since majority of Emirati patients with CIS harbored a heavy lesion load, as per DIS and DIT criteria at the onset of their disease, and met the criteria for CDMS. Additionally, most of the MS patients were younger, educated, and raised in western lifestyles favoring awareness and early access to healthcare, rather than presenting with delayed diagnosis.

In summary, onset disease characterization of MS in Emirati patients on MR imaging using McDonald's DIS and DIT criteria, showed a high lesion load at the onset. This is indicative of hibernating disease from a younger age, with clinical ramifications of early diagnosis, timely commencement of disease modifying therapy, and potentially affecting disability progression. This also enhances the understanding of the natural course of MS in a region which has now become a moderate-risk zone for the disease.

## Figures and Tables

**Table 1:** Onset disease dissemination in space (number of zones involved).

DIS criteria	Percentage
1 out of 4	<1% (1)
2 out of 4	15% (7)
3 out of 4	35% (16)
4 out of 4	49% (23)

**Table 2:** Onset disease dissemination in space (types of zones involved).

DIS zone	Percentage
Periventricular (1)	100% (47)
Callosal/CS interface (I)	85% (40)
Anterior temporal PV (I)	76% (36)
Sub-cortical/Juxta-cortical/Cortical (II)	79% (37)
Infra-tentorial (III)	72% (34)
Cervical spinal cord (IV)	80% (36/45)

**Table 3:** Periventricular zone (PVZ) dissemination in space.

	Percentage
<3	4% (2)
3–5	19% (9)
6–9	17% (8)
7–9	15% (7)
>12	45% (21)

## Data Availability

The data collected in this study is preserved and can be available and can be shared if needed.
